# Saprolegniosis in Amphibians: An Integrated Overview of a Fluffy Killer Disease

**DOI:** 10.3390/jof8050537

**Published:** 2022-05-22

**Authors:** Sara Costa, Isabel Lopes

**Affiliations:** CESAM & Department of Biology Campus of Santiago, University of Aveiro, 3810-193 Aveiro, Portugal; ilopes@ua.pt

**Keywords:** amphibians, disease, water mold, *Saprolegnia*, oomycetes, host, pathogen

## Abstract

Amphibians constitute the class of vertebrates with the highest proportion of threatened species, with infectious diseases being considered among the greatest causes for their worldwide decline. Aquatic oomycetes, known as “water molds”, are fungus-like microorganisms that are ubiquitous in freshwater ecosystems and are capable of causing disease in a broad range of amphibian hosts. Various species of *Achlya* sp., *Leptolegnia* sp., *Aphanomyces* sp., and mainly, *Saprolegnia* sp., are responsible for mass die-offs in the early developmental stages of a wide range of amphibian populations through a disease known as saprolegniosis, aka, molding or a “*Saprolegnia*-like infection”. In this context, the main objective of the present review was to bring together updated information about saprolegniosis in amphibians to integrate existing knowledge, identify current knowledge gaps, and suggest future directions within the saprolegniosis–amphibian research field. Based on the available literature and data, an integrated and critical interpretation of the results is discussed. Furthermore, the occurrence of saprolegniosis in natural and laboratory contexts and the factors that influence both pathogen incidence and host susceptibility are also addressed. The focus of this work was the species *Saprolegnia* sp., due to its ecological importance on amphibian population dynamics and due to the fact that this is the most reported genera to be associated with saprolegniosis in amphibians. In addition, integrated emerging therapies, and their potential application to treat saprolegniosis in amphibians, were evaluated, and future actions are suggested.

## 1. Introduction

Pathogens play an important role in the dynamic of wildlife populations [1,2]. They were identified as a major cause for the rapid worldwide decline that is occurring in the populations and species of the class of Amphibia. Actually, there are several published works reporting that pathogens have led populations and/or species into extinction (presently, 41% of the world’s amphibian species are considered threatened) [3,4,5,6].Some of these pathogens that are considered most threatening for amphibians are responsible for several of the diseases, infections, and infestations registered in the OIE-List of 2021 for amphibians, including the chytrid fungi *Batrachochytrium dendrobatidis* (Bd), *B. salamandrivorans* (Bsal), and the Ranavirus species [7,8,9]. The World Organization for Animal Health (which has kept its historical acronym OIE [Office International des Epizooties]) provides an annual list of diseases to be considered in the OIE list. These diseases must meet certain criteria, namely, “(1) international spreading of the pathogen (via live *animals* or their products, *vectors,* or fomites); (2) at least one country is demonstrated to be country- or zone-free from the disease in susceptible aquatic animals; (3) a precise case definition is available, and reliable means of detection and diagnosis exist; (4) (a) natural transmission to humans has been proven, and human infection causes severe consequences; or (b) the disease has been proven to cause a significant impact on the health of domestic animals, at a country- or zone-level, considering the occurrence and severity of the clinical signs, including direct production losses and mortality; or (c) the disease has been proven to have a significant impact on the health of wildlife, for instance through the occurrence and severity of the clinical signs, including direct economic losses and mortality, and any threat to the viability of a wildlife population [10]”.

Although these diseases appear on the OIE list, others may occur with equal frequency in amphibians and cause them adverse effects. Namely, aquatic oomycetes (known as “water molds”) that are ubiquitous in freshwater ecosystems may affect a broad range of hosts, including amphibians [11]. They comprise organisms belonging to the genera *Saprolegnia*, *Achlya*, *Leptolegnia*, and *Aphanomyces*, which are among the most harmful genera for fish and crustaceans [12]. Some examples can be given, such as *Aphanomyces astaci*, which causes the disease known as crayfish plague, and *A. invadans*, which causes epizootic ulcerative syndrome; both of these examples are OIE-listed diseases [10]. Concerning the amphibians, aquatic oomycetes are mostly known to cause the disease saprolegniosis. When compared with other infectious diseases (e.g., chytridiomycosis and ranavirosis), saprolegniosis is less commonly reported as a source of serious disease in amphibians, with few publications reporting related mass infection or mortality. Nevertheless, it has been reported as a disease that is responsible for a high mortality rate in the early developmental stages of amphibians and is being proposed as the main cause of the population decline of the Boreal toad (*Bufo boreas*) and the Cascades frog (*Rana cascadae*) in the Pacific Northwest (USA) [13,14].

Despite all the adverse effects saprolegniosis may cause in natural populations of amphibians, most literature has mainly focused on its high economic impacts in aquaculture [12]. Saprolegniosis is sometimes considered synonymous of dermatomycosis, molding, or just “*Saprolegnia*-like infection”. Most studies indicate that the genus *Saprolegnia* is the causative agent of saprolegniosis. However, few papers suggest that close relatives in the family *Saprolegniaceae* may also be influential in causing this disease. Considering that *Saprolegnia* is the most reported genus associated with saprolegniosis in amphibians, and because of its ecological importance on amphibian population dynamics, it will be the focus of this review. In addition, *Saprolegnia* species are considered broad-spectrum pathogens, and special attention must be given to them because most species that cause diseases in fish are also pathogenic to amphibians [14,15].

In the light of disease ecology, host–pathogen interactions change within the context of environmental factors. For instance, some studies have predicted that global warming can change the distribution of hosts and pathogens, which may promote an overlap or segregation in their geographic distribution that, consequently, will lead to an increase or decrease in the transmission of diseases [1]. Furthermore, other case studies have predicted that changes in infection factors and prevalence of the disease may also occur as a consequence of global warming [1,16]. In any case, the disease will depend on which host, or pathogen is more vulnerable to environmental changes. Therefore, following this unpredictable dynamic, an immediate need to compile all that is known about wildlife diseases affecting biodiversity emerges [17].

The main objective of the present review was to bring together updated information about saprolegniosis in amphibians in order to integrate the existing knowledge, identify existing knowledge gaps, and suggest future directions within the saprolegniosis–amphibian research. This review will mainly focus on literature related to amphibians; however, due to the lack of specific literature relating saprolegniosis to amphibians, information on saprolegniosis in fish will also be addressed to strengthen knowledge of this disease.

## 2. Amphibian-Related Oomycetes Phylogeny, Taxonomy, and Genomics

Oomycete species are largely recognized as plant pathogens [18]. However, they reside within a group with high diversity in terms of lifestyles, pathogenicity, and host ranges, which makes them a major concern in terms of food security, economy, natural ecosystems, and wildlife. In this context, oomycete species have become of great interest to researchers, especially due to the exponential increase of genome sequencing and new insights into their biology, evolution, genome organization, and metabolism within the oomycete class.

Based on the existing scientific literature, it is perceivable that the occurrence of saprolegniosis in amphibians is often attributed to a generic “water mold” infection without proper identification being made as to the species responsible for the infection [19,20,21,22,23,24,25,26,27]. In most cases, when researchers attempt to identify the most probable infectious agent, taxonomic identification is based only on the morphological features of the isolates. This classification is based on criteria such as non-septate (coenocytes) hyphae, which are representative of oomycetes (*Saprolegnia* spp., in particular), or on the examination of sexual structures, which are unique to the species but difficult to observed under laboratory culture conditions [28,29].

In more recent works, isolates of oomycetes were analyzed through the use of molecular methods (e.g., restriction fragment length polymorphisms (RFLPs), the internal transcribed spacer (ITS) of the nrDNA [30], or DNA barcoding [31]), allowing their identification at the species level with good accuracy. However, this is a point that should be revised and standardized to facilitate a comparison of the data. Different authors adopt different genes or markers to identify this group of organisms, and ITS was already suggested as a reliable universal barcode for Oomycota [28,32]. Recently, McGowan and Fitzpatrick [33] reviewed the latest advances in genomics of the oomycete class. In this section, we will focus only on oomycetes that are currently known to be capable of infecting amphibians, which mainly belong to the *Saprolegniales* order. Accordingly, *Saprolegniales* is one of the four crown orders (jointly with *Peronosporales*, *Pythiales*, and *Albuginales*) of the oomycete class; this class includes animal and plant pathogens. Genera belonging to the *Saprolegniales* order include *Achlya*, *Aphanomyces*, *Saprolegnia*, and *Thraustotheca* [33]. Relative to *Leptolegnia*, *Aplanes*, and *Newbya*, they still comprise the less reported and investigated groups, and on several occasions have not been included in the analysis of the *Saprolegniales* order. Molecular phylogeny has shown that oomycetes are unrelated to true fungi, at times being relocated as Heterokonta, which are closely related to organisms such brown algae.

The molecular systematics of the Oomycota is still undergoing study and revision [34]. For example, for *Saprolegnia* spp., there are misassigned species names in the nrDNA ITS GenBank sequences, which demand a need for a consensus in the genetic identification of the different species. Hulvey et al. [35], based on morphological and DNA sequence data (ITS and 28S), suggested 10 clades within the *Saprolegnia* genus [35]. Afterward, the work published by Sandoval-Sierra et al. [36] resulted in 18 confirmed species and 11 potentially new species as defined by Molecular Operational Taxonomic Units (MOTUs). More recently, Magray et al. [28], after performing an extensive review of the available literature, reported the existence of 31 species of *Saprolegnia*. Therefore, establishing protocols and methodologies for the univocal species identification of this genus and of other *Saprolegniales* is of the highest importance for future research, to create a solid base for applications such as that of the “type species” definition already used in other microorganisms.

**Figure 1:**
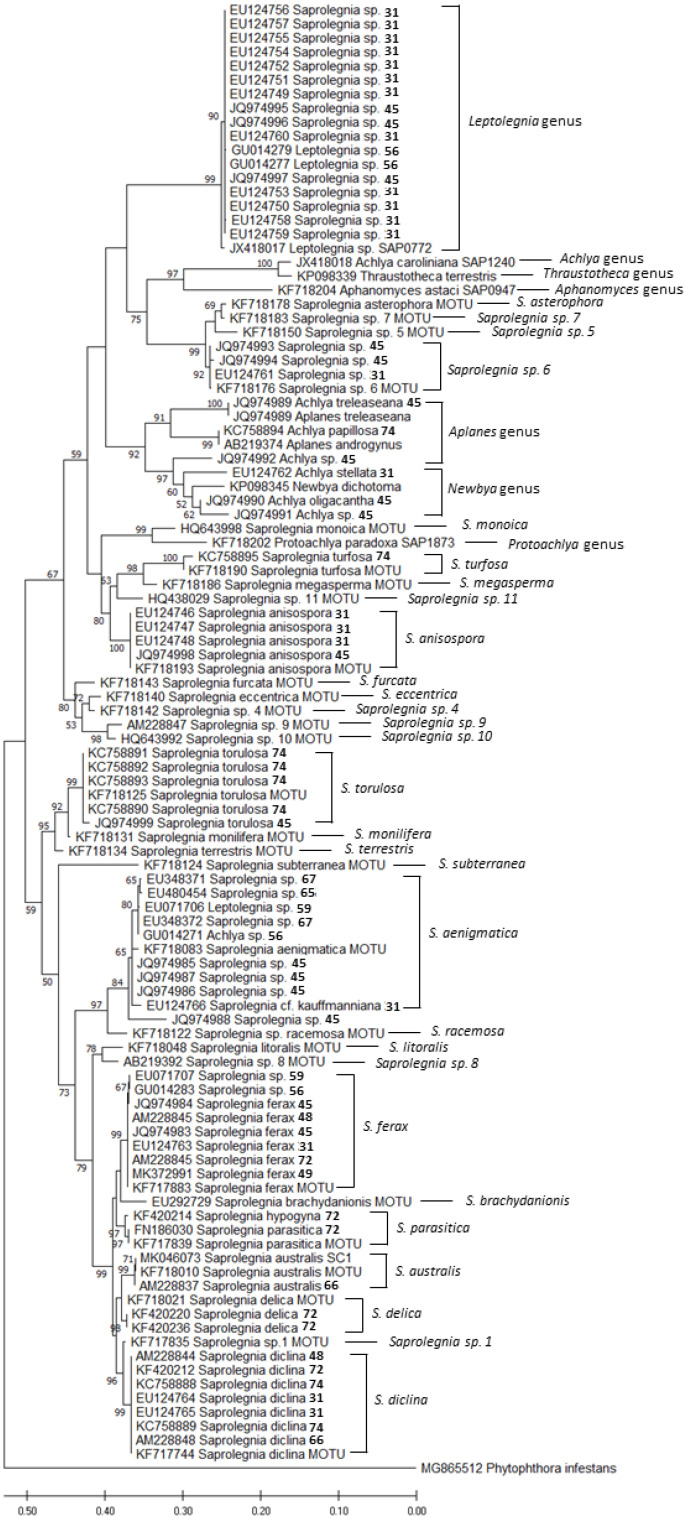
Maximum likelihood phylogram depicting phylogenetic relationships inferred among 105 isolates, based on combined analysis of ITS DNA sequence data. The terminal number corresponds to the bibliographic reference whose sequence belongs in Table 1. Bootstrap values (>50%) are presented to the right of each node.

Aiming for comprehensive molecular information on the status of amphibian-related oomycete species, we compiled all the sequences based on phylogenetic analyses from NCBI (Table 1, Figure 1). A total of 67 different identifications were recovered as FASTA sequences from GenBank, which were analyzed using MEGA X [37] and Clustal W programs. Furthermore, comparison of ITS FASTA sequences was used as a tool for devising a phylogenetic tree, with *Phytopthora infestans* as an out-group. The verification of the accuracy of the sequences identified in the works described in this review was made possible by performing a phylogenetic analysis of the samples by the maximum likelihood (ML) method, along with those suggested by Sandoval-Sierra et al. [36] as MOTUs. The alignment was tested for sequence analyses using default settings, and the robustness of the trees was evaluated by 1000 bootstrap replications. Furthermore, a tree-based analysis on MOTUs was performed using the sequences used in Magray’s review and the amphibian-related sequences identified on the Sandoval-Sierra isolates collection (Appendix A in the Sandoval-Sierra review, [36]). These data are given in the Supplementary Data (Figure S1).

A total of 105 ITS sequences were compared (Figure 1). We identified sequences in which the original name did not match with the closest MOTU (Table S1). The most common mismatch was between *Saprolegnia* spp., identified as *Achlya* sp. or *Leptolegnia* sp., and vice versa. Another problem encountered was the lack of species identification, which was probably due to the species that have not been yet described. This and other problems were extensively analyzed in the review by Sandoval-Sierra et al. [36]. According to the results of the analysis we performed in the present work, 40% of the sequences have inaccurate assignments, and 18% are “incomplete”, without species specification. These results are in line with the findings of Sandoval-Sierra et al. [36], who found that a total of 44% of the whole *Saprolegnia* sequences available in the GenBank had incorrect assignments of species names.

In addition, after an integrative analysis of all the sequences of *Saprolegniales* associated with amphibians, and by using the suggested MOTUs and the *Saprolegnia* spp. list from Magray et al. [28], we found potential incongruencies in the Magray’s ITS tree; thus, the authors suggest caution when choosing sequences for species identifications.

In addition to its application regarding these and other taxonomic challenges, genomics is a revolutionary tool that allows access to finer-scale information, providing a powerful means for studying evolution and enabling, for example, the teasing apart of often-subtle differences among species.

The *S. parasitica* was the first animal pathogen of the oomycete group with its genome to be completely sequenced, followed by *Achlya hypogyna*, *Aphanomyces* spp., and *S. diclina*, all identified as potential agents of saprolegniosis in amphibians [80]. Whilst there is a level of commonality between the biology of the animal and plant pathogenic oomycetes, we are unaware of any oomycete species that causes disease in both plants and animals. It is, therefore, possible that mechanisms of infection have evolved differently to combat the specific threats of the immune responses of different hosts. For instance, the analysis of the *S. parasitica* genome assembly revealed a large divergence comparatively to plant pathogenic oomycetes, showing a lack of RxLRs, CRNs, and NLPs, as well as proteins involved in plant cell wall breakdown (e.g., cutinases) [33]. On the other hand, *S. parasitica* shows a large arsenal of proteases that are differently expressed along the infection stages [80,81].

## 3. Saprolegniosis in Amphibians: From Natural Populations to Laboratory Assays

In the present work, a literature review was performed at the Web of Science (WoS) database to retrieve the papers published under the topic of saprolegniosis in amphibians. The search was made using the strings (amphibian* AND (saprol* OR mould OR mold OR oomycet*)) for the period between 1950 and 2020, during which peer-reviewed articles about saprolegniosis in amphibians were retrieved. In addition, other works, those not appearing in WoS but cited on the reference list of WoS-retrieved papers, were also considered. Information on all study locations, species, and life stages within each article was compiled (Table 1). 

**Figure 2:**
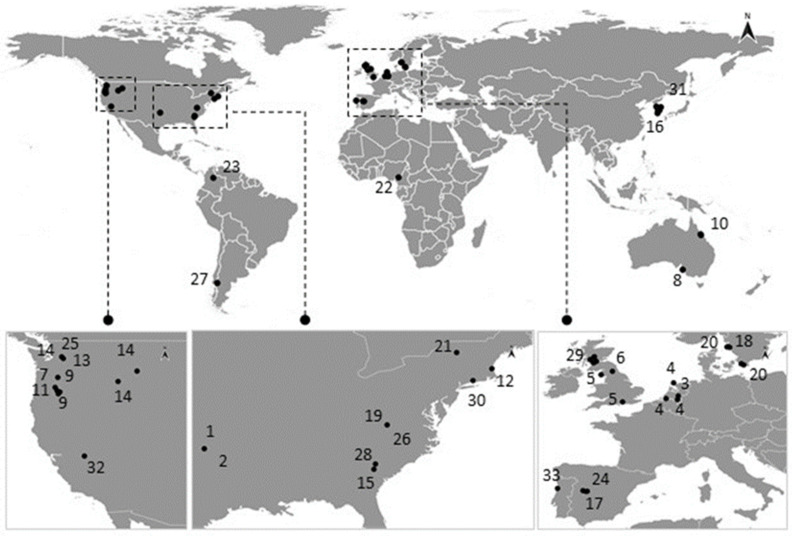
Map showing the geographic distribution of saprolegniosis reports in wild populations. Please see more information in Table 1. Numbers correspond to article reference: (1) Bragg and Bragg [71]; (2) Bragg [56]; (3) Strijbosch [21]; (4) Leuven et al. [22]; (5) Banks & Beebee [23]; (6) Beattie et al. [69]; (7) Blaustein et al. [43]; (8) Williamson & Bull [52]; (9) Kiesecker & Blaustein [44]; (10) Berger et al. [51]; (11) Kiesecker et al. [14]; (12) Gomez-Mestre et al. [41]; (13) Johnson et al.[31]; (14) Petrisko et al. [61]; (15) Ruthig [68]; (16) Kim et al. [67]; (17) Fernández-Benéitez et al. [48]; (18) Sagvik et al. [63,64]; (19) Ruthig [60]; (20) Uller et al. [65]; (21) Karraker & Ruthig [66]; (22) Blackburn et al. [24]; (23) Prada-Salcedo et al. [39]; (24) Fernández-Benéitez et al. [49]; (25) Ault et al. [46]; (26) Ruthig and Provost-Javier [57]; (27) Perotti et al. [59]; (28) Croshaw [75]; (29) Muir [70]; (30) Urban et al. [74]; (31) Groffen et al. [50]; (32) Sadinski, Gallant, and Cleaver [38]; (33) Costa et al. in prep. Maps made with QGIS. Location information available on Table S2.

A map with the geographic distribution of the reports of saprolegniosis in wild populations was performed using QGIS 3.14 (Geographic Information System) [82], as shown in Figure 2 (raw data is shown in Table S2). Despite the still-existing methodological inaccuracies associated with the identification of the pathogens causing saprolegniosis in amphibians, the genus *Saprolegnia* was the most commonly reported in the recovered studies, corroborating the findings of the literature reviewed by Johnson and Paull [12] (Table 1). *Achlya* sp. and *Leptolegnia* sp. were also described and confirmed to be causative of saprolegniosis in amphibians [57,61]. Saprolegniosis is mostly reported as an “early stage” disease in amphibians, showing higher evidence for infection prevalence in early stages in contrast with relatively low reports made in adults [39,45,67,78]. In terms of general occurrence, we found 44 works reporting water mold infection in amphibians (44 species), of which 35 were in the wild, and 35 were based on laboratorial assays, captivity, and/or investigating its infectiveness in complementation with field surveys.

### 3.1. Occurrence in Natural Populations

A total of 38 species of amphibians was reported to be affected by saprolegniosis in the wild. However, considering that several works reported more than one species and that the same species is reported in different works, we considered a total of 65 independent observations (Table S3).

It was also noticed that few works aimed to specifically survey the presence and diversity of water molds associated with natural populations of amphibians. Three works assessed the diversity of *Saprolegniaceae* associated with the eggs of several species of amphibians, in the wild: two in the USA and one in Europe [31,61,70]. A more recent work (in Scotland) specifically investigated the species diversity of the *Saprolegnia* genus associated with and responsible for the occurrence of infection in the eggs of *R. temporaria*; this survey was made across ten locations in Central Scotland [70]. These findings showed that *R. temporaria* eggs inhabiting different pools are subject to infection by different and, sometimes, more than one species of *Saprolegnia*.

The available literature suggests that the occurrence of saprolegniosis in natural populations of amphibians is mostly associated with temperate zones, being mainly reported locally in the USA and in Europe (see Table 1, Figure 2). However, there have also been reports in Australia, Colombia, Argentina, Korea, and Cameroon [39,50,51,59,67] These differential geographic occurrences of saprolegniosis in amphibians may relate to the fact that *Saprolegnia* spp. are reported more often in the USA and Europe, probably because it affects more amphibian populations in these regions (e.g., due to environmental characteristics of these temperate regions) or because there is a lack of information for other regions, which leads to a potential bias that is associated with the published observations.

In addition, it was noticed that the incidence of saprolegniosis has been associated with amphibians present in freshwater ecosystems at higher altitudes [13,70]. Nevertheless, there is only one published work that studied and identified an association between altitude and *Saprolegnia* prevalence in amphibians [70]. Namely, the occurrence of *Saprolegnia* infection in amphibians in South America was identified in populations that were located at altitudes of 780 to 2900 m and, in Cameroon (Lake Oku), at 2227 m [24,39]. We suggest that such high incidences of saprolegniosis in populations of amphibians inhabiting high-altitude habitats may be associated with a simultaneous exposure to increased UV-B intensity. In fact, the potential impact of increased UV-B on the aquatic life stages of amphibians has been vastly reported in scientific literature, including its contribution to immunosuppression, skin damage, mutations, and alteration of associated microbiomes, which may make the organisms more susceptible to other environmental stressors, namely, pathogens [83]. Furthermore, Kiesecker and Blaustein [13] found that *Saprolegnia* sp. and UV-B radiation exposure can act synergistically, decreasing the survival of the embryos of amphibians. This study was performed in situ in two different lakes: the Three Creeks Lake (elevation, 2000 m) and the Lost Lake (elevation, 1220 m). Authors evaluated the hatching success of *R. cascadae*, *B. boreas*, and *Hyla regilla* larvae, which were exposed simultaneously to UV-B radiation and *S. ferax*. In line with this assumption, amphibians most at-risk of UV-B radiation exposure are likely to be those that breed at high altitudes in clear lakes, where the UV-B penetration is higher. For example, some of the published works reporting saprolegniosis are from Lost Lake (Oregon, USA), one of the clearest lakes with the highest transmission of UV-B radiation in the Pacific Northwest [84].

### 3.2. Laboratory Assays

Laboratory experiments (Table 1) constitute the most important and common approach used to study, under controlled conditions, the host–pathogen interaction, allowing for the isolation and manipulation of biotic and abiotic factors. Laboratory challenge assays performed with water molds and amphibians have demonstrated that virulence and infection depend both on the *Saprolegniaceae* and on the amphibian species [47,49,60,63,64]. Perotti et al. [59] observed a differential mortality in the eggs of *Pleurodema thaul*, which was influenced by the *Saprolegnia* species used for the infection experiments. On the other hand, Romansic et al. [62] reported a species dependency on the mortality that *S. ferax* caused in embryos and juveniles of several amphibian species. Furthermore, the different characteristics of the biology of amphibians may also influence the occurrence of saprolegniosis. For example, the behavior involved in laying eggs may have such an influence because a single oviposition can markedly reduce cross-infection of the eggs when compared with a strings or clutches type [44,78]. In addition, virulence assays have demonstrated that a higher incidence of infection occurs during earlier life stages of development, with a tendency toward a decrease in the susceptibility to infection as the development proceeds [14,45,47,62]. For terrestrial developmental stages, this type of information is scarce, but for the species *R. cascadae*, it has been reported that *Saprolegnia* sp. could compromise newly metamorphosed individuals [45]. These challenge assays also served to prove that by adding to their saprotroph characteristics, water molds really act as pathogens in its two main life cycle stages, i.e., zoospores and mycelial spreading [57,60].

An exposure to water molds may also cause sublethal effects in amphibians. Laboratorial assays performed with the embryos of several North American and European anuran species (e.g., *R. sylvatica*, *B. americanus*) reported that exposure to *Saprolegnia* sp. and *Achlya* sp. induced earlier hatching [41,42,59,65]. Though this response potentially helps to avoid infection; it may have a cost on the species’ development. Uller et al. [65] found that tadpoles hatching from clutches that were exposed to *Saprolegnia* sp. had a 20% decreased mass at metamorphosis, even if no further exposure to the pathogen occurred after hatching. However, this topic needs further investigation, since different results are reported in the literature, depending on the amphibian species. For example, Kiesecker and Blaustein [54] found that exposure to *S. ferax* reduced larval recruitment in *R. cascadae* by 46.2%, while no effect was observed on the larval recruitment of *H. regilla*. These authors also reported that the larvae of *R. cascadae* that survived *S. ferax* infection developed faster and were larger at metamorphosis when compared to individuals not exposed to *Saprolegnia*.

The effects of *Saprolegnia* spp. at the sub-individual level have also been reported in the literature. Ghirardi et al. [58] assessed the physiological responses induced by *Saprolegnia*-like sp. on embryos of *Physalaemus albonotatus*. In addition to increased mortality and earlier hatching rates (in line with results reported for other amphibian species), these authors also evaluated the effects on four oxidative stress markers: glutathione reductase (GR), catalase (CAT), lipid peroxidation (LPO), and glutathione S-transferase (GST). Regarding these biochemical markers, only CAT was significantly inhibited in the embryos exposed to the water mold treatment when compared to embryos from the control group. No significant effects were observed for the activity of the other studied enzymes or for LPO levels [58]. More recently, the same author referred to *Saprolegnia*-like species as a genuine stressor, altering the physiological state of the embryos of other species such as *Elachistocleis bicolor.* Among antioxidant defenses, the activity of superoxide dismutase (SOD) and GST increased in embryos exposed to *Saprolegnia*-like species. However, no difference in LPO levels was found between treatments, which might indicate that SOD and GST activation could be sufficient to prevent oxidative damage. Finally, they found a higher mortality and number of malformations in the water mold treatment group [27].

Experiments manipulating environmental conditions have yielded results pointing to the conclusion that, at least in the tested species (e.g., *B. boreas*, *H. regila*, and *R. cascadae*), a high UV-B incidence and a low water temperature during breeding season are associated with a higher incidence of *Saprolegnia*-infected clutches, which could lead to seasonal and geographic variation in the degree of infection [23,69]. Sagvik et al. [63] investigated the effects of family and population variation on the intensity of embryonic infection by *Saprolegnia* sp. For this, the authors analyzed *R. arvalis* eggs from six populations that were exposed to two different temperatures (15 and 18 °C) and observed more infected eggs and a higher mortality rate in organisms that were exposed to the lowest temperature.

Finally, several works tried to corroborate the hypothesis of other taxonomic groups, such as fish, acting as active vectors or reservoirs of saprolegniosis agents, highlighting the inter-class transference of the disease [14,53]. Transmission experiments exposing eggs of *Engystomops petersi* to *S. diclina*-infected rainbow trout (*Oncorhynchus mykiss*), showed 85% mortality when compared to 31% in the control (rainbow trout non-infected) [53].

## 4. An Oomycete Called *Saprolegnia*

Based on the literature review of the previous section (Table 1), *Saprolegnia* genus is identified, so far, as the most reported agent responsible for the occurrence of saprolegniosis in amphibians, with a special focus on *S. ferax* and *S. diclina*. *Saprolegnia* sp. belongs to the *Oomycota* (*Straminipila*), which is a group of fungus-like protists that shares morphologic and ecological features with true fungi such as filamentous growth, life cycle, and osmotrophic feeding [85]. Fungi and oomycetes play a pivotal role in the cycling of organic matter in aquatic ecosystems. Masigol et al. [86] showed that oomycetes and fungi may have complementary roles on the types of biomolecules degraded and substances produced. Fungi degrade complex polymers more effectively and have a marked role in the production of humic substances (HS); oomycetes play a major role on the degradation of small organic molecules, having difficulty in the degradation on more complex polymers. This specificity for the degradation of smaller molecules reveals the opportunistic behavior of oomycetes comparatively to the fungi [86]. Despite their biologic similarities, the phylogenetic detachment of the fungi and oomycetes is reflected in the differences in their biochemistry, cell structure, and development [87].

### Saprolegnia: A Primary or Opportunistic Pathogen?

It is not consensual in the literature whether *Saprolegnia* sp. is a “primary” or “opportunistic” pathogen since it is difficult to define whether the infection occurs before or after the death of the host; however, it can, in fact, act as both [43,48]. For example, *S. australis* is capable of infecting living and apparently healthy *Pelophylax perezi* tadpoles (personal observation). Other works reported that zoospores of *S. diclina* and *S. ferax* are capable of infecting both dead and living healthy eggs. Blaustein et al. [43] and Fernández-Benéitez et al. [48] reported healthy embryos of *R. pipiens* and of *B. terrestris* infected by *S. ferax* and *S. diclina*, respectively. In line with this, the *Saprolegnia* genus consists of opportunistic biotrophic or parasitic pathogens of various aquatic life stages of amphibians and of fully aquatic amphibian species. For example, Songe et al. [88] suggested that *S. diclina* and *S. parasitica* could employ different infection strategies when colonizing and infecting the eggs of Atlantic salmon. The former is capable of destroying the chorion of eggs, acting necrotrophic, while *S. parasitica* hyphae can penetrate an apparently intact chorion, suggesting a biotrophic facultative strategy. Similarly, in experiments performed with *Saprolegnia* sp. growing over eggs of *Ambystoma maculatum* with no jelly coat, the mold could easily penetrate the egg capsule, with hyphae reaching and killing the embryos without any general degradation of the egg capsules [41].

**Figure 3:**
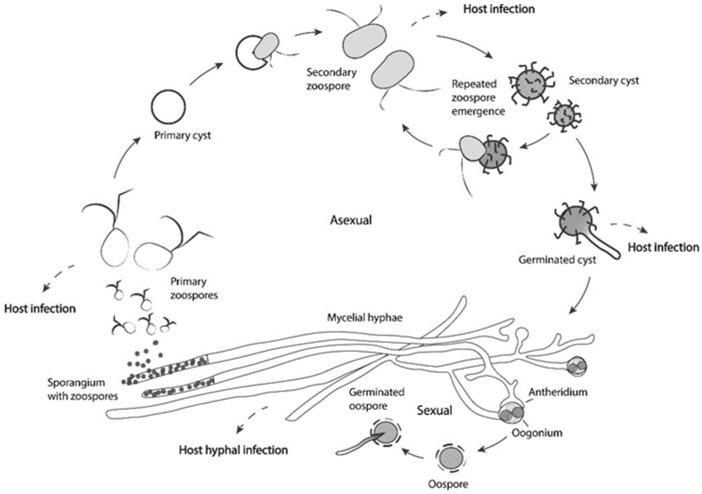
Scheme illustrating the sexual and asexual lifecycles of *Saprolegnia* spp. with the identification of the development stages able to cause infection in amphibians (host infection).

Adding to these facts, *Saprolegnia* sp. hold specialized structures and have evolved life cycle strategies that facilitate host colonization, potentiating their role as a pathogen. *Saprolegnia* sp. are capable of both sexual and asexual reproduction (Figure 3). Although it is not often reported in *Saprolegnia* spp., the sexual stage of the lifecycle is used to enhance survival and fitness during adverse environmental conditions, creating oospores that will germinate when conditions become more favorable [29]. On the other hand, asexual spores are assumed to be important in proliferation. The asexual spores are formed at the end of hyphal cells, the sporangia, which can release many motile zoospores. Usually, a decrease in available nutrients or a sudden drop in environmental temperature is known to trigger zoospore formation [89]. These primary zoospores swim for a short period; afterward, they encyst and release secondary zoospores that are motile for a longer period and are considered the main dispersion and infection structures that are responsible for saprolegniosis [88,90]. After new encystment, the cysts can release a new zoospore. This repeated zoospore emergence (RZE) and encystment is called “polyplanetism” [91].

Zoospores are attracted by chemotaxis to the host and have specialized structures that can potentiate their infection capacity [92,93,94]. Recent works have shown that *S. parasitica* secondary zoospores have longer hook structures when compared to other *Saprolegnia* sp., and that the strength of the zoospore attachment is proportional to the hook length, which can explain its virulence [90,95].

Furthermore, genome and secretome exploitation in some species of *Saprolegnia* unveil specific characteristics and machinery that reinforce the idea that *Saprolegnia* is an active pathogen (see Section 6). *Saprolegnia* has the capacity to digest proteins of living animal hosts, as well as the ability to uptake and catabolize amino acids from an environmental mixture [96].

## 5. Factors That Can Influence Saprolegniosis

Key environmental parameters such as temperature, chemical contamination, and predation, among others, have a profound role in impairing the amphibian immune system and can cause a decrease in individual fitness and compromise population recruitment (reviewed by Rollins-Smith [97]). Similarly, such environmental parameters may affect water molds in diverse ways [15]. Though studies have been carried out to establish an association between specific biotic and abiotic factors and the occurrence of saprolegniosis in amphibians, such an association is still difficult to establish and requires further scientific study.

### 5.1. Saprolegnia sp. as Infectious Agent

Few studies have specifically investigated the influence of environmental parameters on *Saprolegnia* sp. Koeypudsa et al. [98] investigated the effects of pH, temperature, and sodium chloride (NaCl) on several isolates that were identified as belonging to the genus *Saprolegnia*. These authors reported that the optimum pH, temperature, and % (*w*/*v*) NaCl concentrations for growth were 7 to 10, 25 °C, and 0 to 0.5, respectively. However, their data showed that, despite the tested isolates being capable of growth on a wider range of pH (4 to 11), temperature (5 to 30 °C), and % (*w*/*v*) NaCl concentrations (0 to 2.5), vegetative growth was lower than that reported in optimum intervals, which indicates that some intensities of these environmental parameters can negatively affect the growth of *Saprolegnia* [98]. For example, a decrease of the optimum growth temperature by 5 °C caused a reduction of 12% in the mycelial growth of the tested strain, while an increase of 5 °C caused 66% of inhibition on vegetative mycelia radial growth.

Other factors, such as water hardness, may also influence the growth rate of *Saprolegnia* sp. and, consequently, its colonization behavior. Barnes et al. [99] showed that the growth rate of *S. diclina* was clearly influenced by changes in water hardness (either by calcium sulphate dihydrate or magnesium sulphate heptahydrate). The results obtained by these authors showed that *S. diclina* readily colonized seeds of the hemp *Cannabis sativa* (used as a surrogate of egg fish) in solutions with a water hardness above 300 mg/L and even as high as 1200 mg/L. Below hardness values of 150 mg/L, the mold took twice as long to attach to the hemp seeds and to achieve the same growth rate as compared to 300 mg/L.

The zoospore production and their activity may as well be influenced by a variety of environmental variables. Increasing the water temperature from 5 to 25 °C causes an increase in the number of released zoospores, with no apparent reduction in the activity [92]. The optimum temperature for zoospore release corresponds to 20 °C, with a maximum production of zoospores that are 400 and 100% superior to those released at 10 and 15 °C, respectively. Zoospores are produced and maintain normal activity over a wide range of pH values; however, the vast majority are produced at pH 7. At pH values above or below seven, a reduction of at least 65% of the number of produced spores may occur for *S. diclina* [92]. Zoospore production and activity are also markedly influenced by oxygen tension (mmHg), with a considerable reduction in the number of zoospores released at oxygen tensions below 51 mmHg [92].

This kind of knowledge is useful and most relevant for predicting the occurrence, epidemiological surveys, and distribution of *Saprolegnia* sp. in amphibian health monitoring.

### 5.2. The Amphibian Hosts

The scientific literature shows that saprolegniosis in amphibians, mainly in those kept in captivity, strongly correlates to the level of water quality. In captivity, inadequate housing and handling conditions are two of the main reasons for the appearance of saprolegniosis [100]. The most frequent incidence in adults is caused by secondary infection due to bites, abrasion, or small wounds, or is secondary to other infections [73].

Factors predisposing eggs to saprolegniosis include the decomposition of infertile eggs, early embryonic death, and trauma (physical or chemical) to the protective egg membranes and capsule(s) [47]. Robinson et al. [47] demonstrated that dead eggs are readily infected by *Saprolegnia* sp. They also showed that disturbance to the jelly capsules may be detrimental to the embryo’s normal development, potentiating infection by *Saprolegnia* sp. Furthermore, these authors reported that contact infection between adjacent amphibian eggs seemed to be an important factor regarding the spread of the infection [47].

When infection in early stages is widely spread (in the wild or in captivity), attention should turn to poor water quality, water-borne chemicals, pollutants, infectious or toxic diseases in the parent’s reproductive tract, or improper incubation temperatures [100]. In the wild, many factors have been suggested to potentiate infections, namely, species ecology and environmental parameters (e.g., pH, temperature).

The season in which eggs are laid (e.g., fewer infection rates occur in spring than in late spring or summer for *P. thaul*), the type of posture (single, clutches), and the specific characteristics of the eggs (e.g., jelly coat) of each species are important factors in determining a lower or higher susceptibility to infection by *Saprolegnia* sp [41,44,59,78]. Saprolegniosis is often referred to as a “winter disease” in fish aquaculture. In line with this, several works have also linked the occurrence of *Saprolegnia* outbreaks in amphibians to low temperatures [64,69]. Such increased occurrences of saprolegniosis in winter or other lower-temperature conditions may be due to the fact that in amphibian reproduction, the early stages of development and survival are highly influenced by temperature and precipitation [101]. For example, for *R. sylvatica*, a species affected by saprolegniosis, warmer winters led to earlier breeding, which in turn was associated with cooler post-breeding temperatures that slowed larval development [101,102]. As mentioned above, *Saprolegnia* sp. is capable of supporting and growing in a large range of temperatures. However, amphibians, as ectotherms, are expected to be affected by temperatures out of their optimum range, which suggests its higher sensitivity to variations in this environmental parameter when compared to *Saprolegnia* sp. and, thus, are more susceptible to infection at low temperatures. The pH values have also been shown to influence the vulnerability of amphibian eggs to infection by *Saprolegnia* spp. [21,70]. Strijbosch [21] investigated habitat selection during the aquatic phase of three frog species (*R. arvalis*, *R. esculenta*, and *R*. *temporaria*) and three toad species (*B. bufo*, *B. calamita*, and *Pelobates fuscus*), which covered a pH interval between 3.7 to 6.3. Authors found that for the studied species, the occurrence of molding in the spawns was, on average, 100% in sites with a pH below 4.5 [21]. On the other hand, Muir et al. [70] investigated the distribution of amphibian-associated water molds in Scotland ponds where *R. temporaria* eggs were present. The pH of the studied ponds ranged from 5.8 to 6.4. The authors found that the acidity was significantly lower at sites where *Saprolegniaceae* were present. These results show that pH may have a role in the eggs’ susceptibility to water molds under natural conditions. However, other factors may influence the role of pH on the eggs’ susceptibility, namely, the type of vegetation and natural characteristics of the ponds. Therefore, assays under controlled conditions are needed to fully determine the role of pH on *Saprolegiaceae* infection.

Increased exposure to UV-B radiation, resulting from lower water levels, acts synergistically with the water mold *Saprolegnia ferax* to reduce embryonic survival in amphibians in the Western United States [13,14]. In this experiment, embryos were exposed to three sunlight regimes (unfiltered sunlight, sunlight filtered to remove UV-B and shorter wavelengths, and sunlight filtered to remove wavelengths shorter than UV-B), plus natural densities of *Saprolegnia*, which were added artificially to the test organisms. Results showed that UV-B radiation and *S. ferax* density influenced the hatching success of *B. boreas* and *R. cascadae*, with a decrease of about 10 to 20% [13]. 

Another factor that often appears in literature that is associated with the occurrence of saprolegniosis in amphibians is altitude [49,70]. It must be highlighted that high-altitude habitats are frequently characterized by higher UV-B radiation exposure, which can increase water mold infection [13,14].

A few other studies that involved testing the synergism hypothesis between different types of habitat contamination and an increased susceptibility to disease revealed a lack of interactions between these factors, that were less than additive interactions [55,66,76,103]. For example, road de-icing salt (the principal component of which is sodium chloride) in the presence of water molds showed a lack of interaction in influencing the survival of amphibian embryos [66]. Exposure to nominal concentrations of 5 and 20 mg/L of sodium nitrate was shown to decrease *Saprolegnia* sp. infection in *R. aurora* larvae, while silt exposure increased the susceptibility of *Ambystoma tigrinum* to the water mold *S. parasitica* [55,76].

The disruption of the amphibian’s skin microbiome may also explain several cases of saprolegniosis, and in fact, this hypothesis was already used for *Bd* [104]. The hypothesis on the protective role of the amphibian skin microbiome against saprolegniosis-causative agents is reasonable; firstly, the presence of beneficial bacteria against *S. parasitica* in fish [105] has already been shown, and there are frog skin bacteria capable of inhibiting *Saprolegnia* sp. in vitro (personal contribution). So, alterations of skin microbiome function and composition or beneficial cutaneous secretions against pathogens could be related to changes in the host (e.g., alimentation or stress) or in the environment [106,107].

## 6. Pathogenesis and Clinical Signs

Little is known about the pathogenesis of *Saprolegnia* spp., specifically to amphibians. Most of the available information is based on information from animal pathogenic oomycetes (*Saprolegnia* included) but is mostly based on information transposed from fish studies or genomics. Beyond the biological characteristics of the pathogenic agent (discussed on Section 4) data have revealed a diverse ensemble of substances segregated by this mold that support, for example, the adhesion to the host, and that actively contribute to their infection success and virulence [80,90]. As another example, the analysis of small sets of expressed sequence tag data of *S. parasitica* (fish and amphibian pathogen) revealed the segregation of a diverse assemblage of proteins with a potential role in virulence, such as glycosyl hydrolases, proteases, and protease inhibitors, as well as of proteins involved in protection against oxidative stress. Animal pathogenic oomycetes cause infection primarily through the secretion of extracellular enzymes, combined with mechanical pressure activities of their mycelia; in addition they secrete effector proteins that disrupt host cell counterparts, modulating host immune responses and triggering host necrosis [80,81]. Example, the *S. parasitica* data set included a host-targeting protein SpHtp1. It was also shown that *S. parasitica* is capable of downregulating adaptive immune response in fish, maybe due to the production of exogenous prostaglandin E2. Fish demonstrate a strong activation of the innate immunity (e.g., antimicrobial peptides β-defensin 3, cathelicidin-2, and hepcidin highly), as induced by the infection [108].

Considering the impact of saprolegniosis in amphibians, one should take into consideration the main developmental stages of this group of organisms: embryonic, tadpole, and adult. The most-reported effects focus mainly on amphibian eggs, pointing to high mortality levels, which are dependent on environmental factors and on the health conditions (or viability) of the eggs (please see Section 5.2). In addition, water molds typically infect dead or non-fertilized eggs and then start to spread, compromising all the spawn, and increasing the eggs’ mortality [43]. In infected eggs, one of the signs of infection is the presence a thin layer of white fuzz over the surface of the jelly. Water molds may also act as primary skin or oral pathogens during amphibian larval stages. Clinical signs of saprolegniosis in larval stages of amphibians include the external appearance of fungal colonies that appear as wet and cotton-like, erythematous, or ulcerated skin. Although infections generally affect the tail, hindlimbs, gills, and oral mucous membranes without becoming systemic, lesions sometimes deeply penetrate and involve underlying tissues [100].

In the adults of amphibians, analysis of fungal-infected tissues or lesions with a wet, cotton-like appearance often reveals mats of aseptate, sparsely branching fungal filaments. Any inflammatory response is generally minimal, but if the lesions are not treated immediately, the hyphae will rapidly spread, and the areas near the infection will start to show ulceration, necrosis, and/or oedema, depending on the severity [19,20,72]. In salamanders, additional signs can include anorexia, weight loss, lethargy, vomiting, and respiratory distress [20]. In severe cases, death can occur, presumably from osmoregulatory impairment [109].

## 7. Treatment and New Therapies

### 7.1. Treatment

Several treatments have been used to treat saprolegniosis in amphibians kept in captivity. However, most of them were adapted from treatments applied to fish species and, therefore, the pharmacologic properties, safety, and often the efficacy of the treatment have not always been properly investigated in amphibians (Table 2). Based on this lack of information, caution should be taken when applying treatments recommended for fish in amphibians since they exhibit important biological differences. For example, tetracycline (a broad-spectrum antibiotic) is widely used in aquaculture and ornamental fish. Based on this, it has also been recommended to treat amphibian bacterial diseases such as the red leg main agent, *Aeromonas hydrophila*. It was found that in *R. pipiens*, the dosage recommended to effectively treat the infection, administered through bathing, may damage the skin [110]. Therefore, caution must be taken when using this antibiotic in amphibians in general. Antibiotics are highly effective in controlling infections; however, they can also alter the natural skin microbiome, allowing opportunistic pathogens to establish [111,112]. In addition, considering the different routes of administration for therapeutic agents (orally, topically, bath, or injection), the effectiveness and the dosage of the treatment should be carefully monitored and individually adjusted to each diagnosis [111,113].

In addition, the following information (Section 7.2) is based on experiments that were performed in captivity, and information about mitigation of saprolegniosis in wild populations is non-existent.

Most of the treatments that are currently applied to treat saprolegniosis in amphibians were developed in the 90s and early 2000s [115]. Case studies and reports of zoos and/or aquariums support the efficacy of these compounds in different amphibian species [100]. Techniques used to control saprolegniosis in amphibians in captivity are based on baths of antifungal products, the most traditional ones being sodium chloride or sea salt. In some cases, it is suggested that the area infected with the growing mycelia should be cleaned, followed by the application of an antifungal ointment. In more severe cases, parenteral antibiotics may be warranted to prevent bacterial infection [100].

Amphibian-skin-damaging diseases such as saprolegniosis can cause, in addition to difficulties in gas exchange, significant electrolyte imbalances that will result in other health complications, especially when the skin is severely damaged [19,130,131]. Therefore, in addition to antifungal treatments, affected animals should benefit from fluid and electrolyte therapy. For mildly to moderately affected adult animals, electrolyte baths, such as amphibian Ringer’s solution, applied continuously to aquatic species or supplied as a water source for terrestrial amphibians may be adequate [132]. In addition, saprolegniosis often occurs at temperatures below 20 °C; thus, an adjunct therapy should include elevating the enclosure temperature above this value if the host amphibian species can tolerate higher temperatures [100].

Despite the prevalence of infections being mainly reported during early developmental stages, treatments for these life stages are rarely reported. Michaels et al. [26] suggest that for *Hynobius* sp. eggs, the most effective treatment is the use of methylene blue (a prophylactic also used in laboratories to prevent fungal infection on eggs of *Danio rerio*) and the separation of the infected eggs.

### 7.2. New Therapies

In vitro studies have suggested potential candidates that may control *Saprolegnia* sp. infections. Ali et al. [120] showed that boric acid (BA) completely inhibits the germination and colonization of *S. parasitica* and *S. diclina* spores in vitro ([BA] = 800 mg/L) and impairs the mycelium growth ([BA] > 200 mg/L). The efficacy of this treatment was studied in vitro with salmon eggs and fry, yielding positive results for the eggs to which 200 to 1400 mg BA/L were applied, and for the fry treated with 500 mg BA/L (both had continuous exposure for 14 days), with no negative impact on hatchability or viability of organisms [119]. However, the efficacy of this treatment in amphibians still needs further investigation since impairments on reproduction was observed in *Xenopus laevis* that were continuously exposed for 30 days to high concentrations of boron (NOAEC (no observed effect concentration) equivalent to 57.0 mg BA/L for reproductive endpoints and 85.5 mg BA/L for fecundity) [133]. Regarding early developmental stages, a study reported that high levels of boron (50 to 100 mg/L) reduced the hatching success of *B. americanus* and produced a high number of deformed offspring in *R. sylvatica*, *Ambystoma jeffersonianum*, and *A. maculatum* [134].

Another promising candidate to treat saprolegniosis in amphibians is clotrimazole. Warrilow et al. [121] observed that this antifungal drug exhibits a high potential for controlling *Saprolegnia* sp. in vitro. They showed a MIC_100,72h_ (minimum inhibitory concentration during which growth remains completely inhibited after 72 h) for clotrimazole of ~1–2 mg/L, which was shown to be as effective at inhibiting *Saprolegnia* sp. growth in vitro as malachite green (MIC_100,72h_~1 mg/L). Its potential adverse effects in amphibians were tested in acute and chronic tests with eggs and tadpoles of *X. tropicalis*, respectively. The authors found that even for dosages below the advanced *Saprolegnia* sp. treatment, clotrimazole showed significant effects on survival, body length, and body mass [135]. Several works advert the potential of endocrine disruption using clotrimazole and other azolic compounds in amphibians (affecting oocyte maturation and ovulation) [136,137]. In addition, clotrimazole is classified as “very toxic to aquatic organisms”, in agreement with the EU directive 67/548/EEC.

For over three decades, Virkon S, a broad-spectrum disinfectant, has been widely used in farming and livestock production. Due to its efficacy against pathogenic bacteria, viruses, and fungi and its relative safety regarding animal and human health, Virkon-S has been authorized by the United Nation’s Food and Agriculture Organization and approved in Korea to secure biosafety and strengthen Emergency Disease Control Contingency Planning. Presently, Virkon S is being tested as a potential treatment agent in a new approach to combat skin disease in fish [122]. It is effective in inhibiting spore release by *S. parasitica* and impairs mycelial growth at concentrations above 4 mg/L. In addition, its potential toxicity was evaluated on *Cyprinus carpio* fingerlings and adults. The results showed no mortality in the fingerlings at concentrations below 10 mg/L after a 96 h exposure period, and treatments of 4 and 10 mg/L proved to be effective in the complete reversal of artificially induced saprolegniosis in the common carp. Moreover, no cytotoxic effect on epithelioma papulosum cyprini (EPC) cells was observed even at a concentration as high as 100 mg/L [122]. Regarding amphibians, Virkon S has been used to prevent the spread of *B. dendrobatidis* among wild and laboratorial populations and to sterilize boots and any field material; it has even been used during housekeeping procedures. In line with this, a few works have investigated its effects on amphibians and found that it did not affect the survival, mass, or the behavior of the common frog *R. temporaria* or common toad *B. bufo* tadpoles [138]. Concentrations ranging from 0.5 to 5 mg/L of disinfectant had no significant effects on *R. arvalis* embryos and hatchlings but did reduce hatching success [139].

Considering the deleterious effects induced by many of the substances that are used in aquaculture to treat disease, alternative methods that use bioactive products from medicinal plants are considered as being more environmentally friendly alternatives. Essential oils (EOs) constitute a group of such alternatives that have been studied actively. Tavares-Dias [140] reviewed the use of EOs, and their major compounds, as treatments against fish parasites, with an extensive revision on the in vivo efficacy, toxicity, and in vitro efficacy for different fish species [140].

In relation to *Saprolegnia* spp., EOs and pure compounds started being tested at least two decades ago, but in recent years have gained special attention [123,124,125,126,127,128,129]. For example, Miljanovíc et al. [126] tested rosemary (*Rosmarinus officinalis*), sage (*Salvia officinalis*), and bay laurel (*Laurus nobilis*) essential oils against *S. parasitica* zoospore. Results showed an EC_50_ for zoospore germination of 0.063, 0.012, and 0.013 μL/mL for rosemary, sage, and bay laurel respectively, against 0.032 μL/mL of malachite green used as a positive control. Also, *Nigella sativa*, *Punica granatum*, *Thymus vulgaris,* and *Zingiber officinale* were tested against *S. diclina* and [125]. Plant extracts of *P. granatum* and T*. vulgaris* were shown to be effective in preventing the mycelial growth of *S. diclina* at a concentration of 0.5 mg/mL. In addition, acute fish toxicity of the plant extracts was characterized by exposing tilapia fingerlings (*Oreochromis niloticus*) to different concentrations of *P. granatum* and *T. vulgaris* extracts for 96 h, and results indicated low acute toxicity [125].

For amphibians, scarce knowledge exists on the use of EOs to combat infectious diseases. In fact, the research of EOs in amphibians is essentially based on their use as anesthetics to reduce distress and pain in animals. For example, Goulet et al. [141] evaluated the toxicity of the phenolic compound eugenol that is used as an anesthetic in *X. laevis*. Eugenol (1-allyl-3-methoxy-4-hydroxybenzene) is the main constituent (about 88%) of the OEs extracted from the dried flower buds of the clove tree *Eugenia caryophyllata*. The authors exposed *X. laevis* females (90–140 g) to a single bath immersion with 350 µg/L of eugenol for 10 min or three consecutive daily administrations of eugenol. After exposure, the individuals were able to recover for 24 h or one week before euthanasia for evaluation of gross lesions and histopathology.

Clove oil (0.3 ml/L immersion) has been shown to be lethal for *Rhinella marina* adults and to cause respiratory depression in adults of *R. pipiens* [142]. Another side effect noted with clove oil in *R. pipiens* (bath immersion < 318 to 350 mg/L) was that 50% of the frogs had a prolapsed stomach after being removed from the clove oil solution [143]. Tiger salamanders, *Ambystoma tigrinum,* require higher doses (450 mg/L) than *R. pipiens* to produce surgical anesthesia and side effects were not reported [143].

Other work, from Salbego et al. [144] exposed *Hypsiboas geographicus* tadpoles to the essential oils of *Aniba rosaeodora* (EOAR), *Lippia origanoides* (EOLO), and *L. alba* (chemotypes citral, EOL-C, and linalool, EOL-L). Results showed no mortality, while all studied EOs were effective sedatives and anesthetics for the tadpoles of *H. geographicus*. Specifically, EOLO revealed a narrow safety range, and its use must not exceed 100 μL/L [144]. This available information once more suggests the need for a refinement regarding the application of EO treatments between fish and amphibians. For example, *Eucalyptus globulus,* when combined with other EOs, inhibits the growth of *S. parasitica* (MIC_100,72h_ = 0.018 μL/mL), but showed moderate toxicity (LC_50,48h_ = 35.98 ± 0.82) to *Oncorhynchus mykiss* fingerlings [124]. In amphibians this has not been tested yet, but Hernández-Gómez et al. [145] found that individuals of the salamander *Batrachoseps attenuates* inhabiting areas with *Eucalyptus* plantations instead of native vegetation showed a lower body condition.

Considering the available knowledge for amphibians, clotrimazole and Virkon S seem the best option when analyzing the benefits and efficacy, because the concentrations used that cause no or a low effect for the host organism are near the concentrations that are capable of treating the disease. Nevertheless, new insights into saprolegniosis treatment in amphibians need further investigation, with a special focus on the potential of EOs applications.

## 8. Future Needs

One issue that emerged during the analysis made in this review is that there remain many knowledge gaps regarding the comprehension of saprolegniosis occurrence and its treatment in amphibians. In this subsection, some points are summarized that are useful in predicting occurrences, in epidemiological surveys, and in the distribution of water molds and amphibian health monitoring without overlooking the understanding of the environmental role in disease.

First, after the analysis of literature on the incidence of saprolegniosis in wild populations of amphibians, it has become clear that there is a need for further studies in other regions of the planet in addition to just those in specific temperate zones, in order to clarify the geographic distribution of the disease and the areas of its major incidence. Therefore, more knowledge is needed regarding the influence of environmental parameters (e.g., pH, altitude, UV-B exposure) on the incidence of saprolegniosis in amphibians to identify areas and populations that are potentially more susceptible to infections by water mold.

The need for an accurate methodology to enable a univocal identification of the saprolegniosis pathogens is consensual as one direction for future research and should involve the creation of a solid database for future applications. Furthermore, despite the existing works, the significant interaction between environmental contamination (de-icing salt, motor oil, nitrate) and amphibian susceptibility to water mold infection is still not well understood; thus, further studies will be needed to predict the causal effects of environmental contamination on the occurrence of saprolegniosis in amphibians. Equally underdeveloped is the understanding of the role of environmental contamination on host susceptibility and how it could affect saprolegniosis agents and their potential for infection and virulence.

Despite the ubiquity of *Saprolegnia* sp. in freshwater habitats, several authors have suggested that some species were introduced locally by aquaculture or invasive species [39]. It must be considered that species reported as amphibian pathogens are also common fish pathogens. From this perspective, the pathogen propagation and multi-host potential for natural amphibian populations are of the greatest relevance. Nevertheless, the propagation and introduction of saprolegniosis in native ecosystems is predicted to continue because fish species are still being introduced around the world for food aquaculture (51%), ornamental fish (21%), sport fishing (12%), and fisheries (7%) [146]. The introduction of fish for sport fishing includes species such as the rainbow trout *Oncorhynchus mykiss* and the brown trout *Salmo trutta* L., which are known as being highly susceptible to saprolegniosis and potentially working as a vector and reservoir [53,147].

Although we do not know the true impact of saprolegniosis on population dynamics and consequently on ecosystems, through the analysis of Table 1 and other information that has been indicated in articles, we know that this disease can cause a decline in amphibian populations and also affects populations that are already in decline. In a detailed analysis, saprolegniosis is already affecting species with an IUCN Red List conservation status classification, including: three species that are critically endangered (CR), two endangered species (EN), four vulnerable species (VU), and two species with the classification of being near-threatened (NT), all of them with a decreasing populational trend. In addition, other eleven amphibian species, although classified as least concern (LC), must not be neglected because they share the decreasing populational trend of many other species.

In addition, species such as chytridiomycosis need specific environmental conditions to become established, and its distribution is not as ubiquitous as oomycetes in general. They are present in almost every aquatic system. However, although there are no mitigation mechanisms for saprolegniosis in wild populations as for chytrid, there is a need to find methodologies to control its impact on affected populations.

Finally, the evaluation of the efficacy and safety of new treatments for saprolegniosis in amphibians is a topic that lacks recent revision or exploitation. For fish, maybe mainly due to economic pressure, the effort in reaching new, safe, and more powerful treatments is increasing, yet for amphibians, even in vitro-tested drugs have still not been validated in vivo.

## Figures and Tables

**Table 1:** Studies reporting the occurrence of saprolegniosis in different life stages of amphibians. Whenever possible, the pathogen is indicated accordingly as identified in the original publication. (s)—identification based on phylogenetic analyses; w—reported in the wild; c—reported in captivity; l—laboratory assays (infection during laboratory assays, or artificially exposed for susceptibility assay or for assessing exposure effects to the pathogen); n.d.—not defined. Note: The scientific name of the host species is indicated as it appears in the original publication. IUCN Red List Category: Least Concern (LC); Near Threatened (NT); Vulnerable (VU); Endangered (EN); Critically Endangered (CR).

Host Species	IUCN Red List Category (Last Assessment)	Population Trend	Life Stage	Pathogen	Country	Reference	GenBank Accession Number
**Anura**							
*Anaxyrus canorus*	EN, 2004	Decreasing	Eggs (w)	*Saprolegnia diclina*	USA	[38]	
*Atelopus mittermeieri*	EN, 2017	Decreasing	Adult (w)	*Saprolegnia* sp.	Colombia	[39]	
*Bombina bombina*	LC, 2009	Decreasing	Eggs (l)	*Saprolegnia ferax* *Saprolegnia parasitica* *Aphanomyces stellatus* *Dictyuchus sterilis* *Leptomitus lacteus* (*)	Poland	[40]	
*Bufo americanus* ^1^	LC, 2015	Stable	Eggs (w) (l)	*Saprolegnia* sp. *Achlya* sp.	USA	[41]	
*Bufo americanus* ^1^	LC, 2015	Stable	Embryo (l)	*Achlya* sp. *Saprolegnia* sp.	USA	[42]	
*Bufo boreas* ^2^	LC, 2015	Decreasing	Eggs (w)	*Saprolegnia ferax*	USA	[43]	
*Bufo boreas* ^2^	LC, 2015	Decreasing	Eggs (l)	*Saprolegnia ferax*	USA	[13]	
*Bufo boreas* ^2^	LC, 2015	Decreasing	Eggs (w) (l)	*Saprolegnia ferax*	USA	[44]	
*Bufo boreas* ^2^	LC, 2015	Decreasing	Embryos (w) (l)	*Saprolegnia ferax*	USA	[14]	
*Bufo boreas* ^2^	LC, 2015	Decreasing	Metamorphs (l)	*Saprolegnia* sp.	USA	[45]	
*Bufo boreas* ^2^	LC, 2015	Decreasing	Egg (w)	*Saprolegnia anisospora* (s) *S. diclina* (s) *Saprolegnia.* sp. 1 (s) *Saprolegnia sp*. 2 (s)	USA	[31]	EU124746 to EU124748 EU124759 to EU124753 EU124761
*Bufo boreas* ^2^ (*)	LC, 2015	Decreasing	Eggs (w)	*Saprolegnia* spp. (s) *Achlya* spp.(s)	USA	[46]	JQ974984 JQ974986 to JQ974990, JQ974992 to JQ974999
*Bufo bufo* ^3^	LC, 2009	Stable	Eggs (w)	*Saprolegnia* (Molding)	Netherlands	[21]	
*Bufo bufo* ^3^	LC, 2009	Stable	Eggs (w)	*Saprolegniaceae*	Netherlands	[22]	
*Bufo bufo* **^3^**	LC, 2009	Stable	Eggs (l)	*Saprolegnia ferax* *Saprolegnia parasitica* *Aphanomyces stellatus* *Dictyuchus sterilis* *Leptomitus lacteus* (*)	Poland	[40]	
*Bufo bufo* **^3^**	LC, 2009	Stable	Eggs (l)	“*Saprolegnia diclina*-*S. parasitica* complex”	UK	[47]	
*Bufo bufo* **^3^**	LC, 2009	Stable	Eggs (n.d)	*Saprolegnia diclina* (s)	Spain	[36]	KF717747 KF717750
*Bufo calamita* ^4^	LC, 2009	Decreasing	Eggs (w)	*Saprolegnia* (Molding)	Netherlands	[21]	
*Bufo calamita* ^4^	LC, 2009	Decreasing	Eggs (w)	*Saprolegniaceae*	Netherlands	[22]	
*Bufo calamita* ^4^	LC, 2009	Decreasing	Eggs (w)	*Saprolegnia* “infestation” (**)	UK	[23]	
*Bufo calamita* ^4^	LC, 2009	Decreasing	Eggs (w)	*Saprolegnia diclina*	Spain	[48]	
*Bufo calamita* ^4^	LC, 2009	Decreasing	Embryo (w) (l)	*Saprolegnia diclina* (s)	Spain	[49]	AM228844
*Bufo calamita* ^4^	LC, 2009	Decreasing	Eggs (n.d)	*Saprolegnia diclina* (s)	Spain	[36]	KF717752
*Bufo gargarizans*	LC, 2018	Stable	Eggs (w)	*Saprolegnia ferax* (s)	Korea	[50]	MK372991
*Bufo marinus* ^5^	LC, 2008	Increasing	Tadpoles (w)	*Aphanomyces* sp.	Australia	[51]	
*Crinia signifera*	LC, 2004	Stable	Eggs (w)	*Water mold infections*	Australia	[52]	
*Elachistocleis bicolor*	LC, 2004	Stable	Embryo (l)	*Saprolegnia*-like sp.	Argentina	[27]	
*Engystomops petersi*	LC, 2017	Stable	Embryo (l)	*Saprolegnia diclina* (s)	Ecuador	[53]	
*Hyla molleri*	LC, 2008	Decreasing	Eggs (n.d)	*Saprolegnia diclina* (s)	Spain	[36]	KF717749
*Hyla regilla* ^6^	LC, 2004	Stable	Eggs (l)	*Saprolegnia ferax*	USA	[13]	
*Hyla regilla* ^6^	LC, 2004	Stable	Eggs (l)(w)	*Saprolegnia ferax*	USA	[44]	
*Hyla regilla* ^6^	LC, 2004	Stable	Embryos (l)	*Saprolegnia ferax*	USA	[54]	
*Hyla regilla* ^6^	LC, 2004	Stable	Embryos (w)	*Saprolegnia ferax*	USA	[14]	
*Hyla regilla* ^6^	LC, 2004	Stable	Larvae (l)	*Saprolegnia* sp.	USA	[55]	
*Hyloscirtus alytolylax*	NT, 2004	Decreasing	Eggs (n.d)	*Saprolegnia* sp. 2 (s)	Ecuador	[36]	KF718069
*Lithobates berlandieri* ^7^	LC, 2008	Stable	Tadpoles (w)	*Saprolegnia* sp.	USA	[56]	
*Lithobates* *catesbeiana* ^8^	LC, 2015	Increasing	Eggs (w)	*Leptolegnia* sp. (s)	USA	[57]	GU014271
*Pelobates cultripes*	VU, 2020	Decreasing	Eggs (l) (w)	*Saprolegnia. diclina* (s)	Spain	[49]	AM228845
*Pelobates cultripes*	VU, 2020	Decreasing	Eggs (n.d)	*Saprolegnia diclina* (s) *Saprolegnia* sp. 2 (s)	Spain	[36]	KF717745/ KF717746 KF717748 KF717753 KF717754 KF717770 KF718050 to KF718054
*Pelobates fuscus*	LC, 2009	Decreasing	Eggs (w)	*Saprolegnia* (Molding)	Netherlands	[21]	
*Pelophylax perezi*	LC, 2020	Decreasing	Eggs (n.d)	*Saprolegnia diclina* (s)	Spain	[36]	KF717751
*Pelophylax perezi*	LC, 2020	Decreasing	Eggs (w) Tadpoles (l)	*Saprolegnia australis* (s)	Portugal	Costa et al. (in prep.)	MK046073
*Physalaemus albonotatus*	LC, 2004	Stable	Embryo (l)	*Saprolegnia* sp.	Argentina	[58]	
*Pleurodema thaul*	LC, 2015	Stable	Eggs (l) (w)	*Saprolegnia ferax* *Saprolegnia* sp. *Saprolegnia diclina*	Argentina	[59]	
*Pleroderma thaul*	LC, 2015	Stable	Eggs (n.d)	*Saprolegnia diclina* (s) *S. australis* (s) *S. anisospora* (s)	Argentina	[36]	KF717781 to KF717785 KF717972 KF718194
*Pseudacris crucifer*	LC, 2015	Stable	Eggs (w) (l)	*Leptolegnia* sp. (s)	USA	[60]	EU071706
*Pseudacris crucifer*	LC, 2015	Stable	Eggs (w) Adult (w)	*Saprolegnia* sp. (s) *Achlya* sp. (s)	USA	[57]	GU014279 GU014283
*P* *seudacris* *regilla*	LC, 2004	Stable	Metamorphs (l)	*Saprolegnia* sp.	USA	[45]	
*Pseudacris regilla*	LC, 2004	Stable	Egg (w)	*Saprolegnia.* sp. 1 (s) *S. cf kauffmaniana* (s) *Achlya stellata* (s)	USA	[31]	EU124762 EU124766 EU124754 to EU124757
*Pseudacris regilla*	LC, 2004	Stable	Eggs (w)	*Saprolegnia diclina* (s) *Achlya* sp.(s)	USA	[61]	
*Pseudacris regilla*	LC, 2004	Stable	Larvae (l)	*Saprolegnia ferax*	USA	[62]	
*Pseudacris streckeri*	LC. 2015	Unknown	Tadpoles (w)	*Saprolegnia* sp.	USA	[56]	
*Rana arvalis*	LC, 2009	Stable	Eggs (w)	*Saprolegnia* (Molding)	Netherlands	[21]	
*Rana arvalis*	LC, 2009	Stable	Eggs (w)	*Saprolegniaceae*	Netherlands	[22]	
*Rana arvalis*	LC, 2009	Stable	Eggs (l)	*Saprolegnia ferax* *Saprolegnia parasitica* *Aphanomyces stellatus* *Dictyuchus sterilis* *Leptomitus lacteus* (*)	Poland Ukraine	[40]	
*Rana arvalis*	LC, 2009	Stable	Eggs (l) (w)	*Saprolegnia* spp.	Sweden	[63,64]	
*Rana arvalis*	LC, 2009	Stable	Eggs (l) (w)	*Saprolegnia* sp.	Sweden	[65]	
*Rana aurora*	LC, 2015	Decreasing	Embryos (w)	*Saprolegnia ferax*	USA	[14]	
*Rana aurora*	LC, 2015	Decreasing	Larvae (l)	*Saprolegnia* sp.	USA	[55]	
*Rana aurora*	LC, 2015	Decreasing	Metamorphs (l)	*Saprolegnia* sp.	USA	[45]	
*Rana aurora*	LC, 2015	Decreasing	Eggs (w)	*Saprolegnia* spp. (s) *Achlya* sp.(s)	USA	[61]	
*Rana aurora*	LC, 2015	Decreasing	Larvae (l)	*Saprolegnia ferax*	USA	[62]	
*Rana cascadae*	NT, 2004	Decreasing	Eggs (l)	*Saprolegnia ferax*	USA	[13]	
*Rana cascadae*	NT, 2004	Decreasing	Eggs (w) (l)	*Saprolegnia ferax*	USA	[44]	
*Rana cascadae*	NT, 2004	Decreasing	Embryos (l)	*Saprolegnia ferax*	USA	[54]	
*Rana cascadae*	NT, 2004	Decreasing	Embryos (w)	*Saprolegnia ferax*	USA	[14]	
*Rana cascadae*	NT, 2004	Decreasing	Metamorphs (l)	*Saprolegnia* sp.	USA	[45]	
*Rana cascadae*	NT, 2004	Decreasing	Eggs (w)	*Saprolegnia* sp. (s) *Achlya* sp.(s) *Leptolegnia* sp. (s)	USA	[61]	
*Rana cascadae*	NT, 2004	Decreasing	Eggs (w)	*Saprolegnia* sp. (s) *Saprolegnia* ferax (s)	USA	[46]	JQ974983, JQ974985, JQ974991
*Rana catesbeiana*	LC, 2015	Increasing	Eggs (w) (l)	*Saprolegnia* sp. (s)	USA	[60]	EU071707
*Rana clamitans*	LC, 2015	Stable	Eggs (w) (l) Embryos (w) (l)	*Saprolegnia* (s)	USA	[66]	EU480454
*Rana dalmatina*	LC, 2009	Decreasing	Eggs (l)	*Saprolegnia ferax* *Saprolegnia parasitica* *Aphanomyces stellatus* *Dictyuchus sterilis* *Leptomitus lacteus* (*)	Ukraine	[40]	
*Rana esculenta* ^9^			Eggs (w)	*Saprolegniaceae*	Netherlands	[22]	
*Rana esculenta* ^9^			Eggs (w)	*Saprolegnia* (Molding)	Netherlands	[21]	
*Rana kl. esculenta* ^10^			Eggs (l)	*Saprolegnia ferax* *Saprolegnia parasitica* *Aphanomyces stellatus* *Dictyuchus sterilis* *Leptomitus lacteus* (*)	Poland	[40]	
*Rana huanrenensis*	LC, 2018	Decreasing	Adult (w)	*Saprolegnia diclina* (s)	Korea	[67]	AM228848
*Rana lessonae* ^11^	LC, 2008	Decreasing	Eggs (l)	*Saprolegnia ferax* *Saprolegnia parasitica* *Aphanomyces stellatus* *Dictyuchus sterilis* *Leptomitus lacteus* (*)	Poland	[40]	
*Rana luteiventris*	LC, 2015	Decreasing	Eggs (w)	*Saprolegnia* sp. (s) *Achlya* sp.(s) *Leptolegnia* sp. (s)	USA	[61]	
*Rana plancyi chosenica* ^12^	VU, 2020	Decreasing	Tadpoles (c)	*Saprolegnia australis* (s)	Korea	[67]	AM228837
*Rana pretiosa*	VU, 2004	Decreasing	Eggs (w)	*Saprolegnia* sp. (s) *Achlya* sp.(s) *Leptolegnia* sp. (s)	USA	[61]	
*Rana ridibunda* ^13^	LC, 2009	Increasing	Eggs (l)	*Saprolegnia ferax* *Saprolegnia parasitica* *Aphanomyces stellatus* *Dictyuchus sterilis* *Leptomitus lacteus* (*)	Poland	[40]	
*Rana* *sphenocephala*	LC, 2021	Stable	Eggs (w) (l)	*Saprolegnia* (s)	USA	[68]	EU348371 EU348372
*Rana sylvatica*	LC, 2015	Stable	Eggs (w) (l)	*Saprolegnia* sp. *Achlya* sp.	USA	[41]	
*Rana sylvatica*	LC, 2015	Stable	Embryo (l)	*Achlya* sp. *Saprolegnia* sp.	USA	[42]	
*Rana sylvatica*	LC, 2015	Stable	Eggs (w) (l) Embryos (w) (l)	*Saprolegnia* (s)	USA	[66]	EU480454
*Rana temporaria*	LC, 2008	Stable	Eggs (w)	*Saprolegnia* (Molding)	Netherlands	[21]	
*Rana temporaria*	LC, 2008	Stable	Eggs (w)	*Saprolegniaceae*	Netherlands	[22]	
*Rana temporaria*	LC, 2008	Stable	Eggs (w)	*Saprolegnia* sp.	UK	[69]	
*Rana temporaria*	LC, 2008	Stable	Eggs (l)	*Saprolegnia ferax* *Saprolegnia parasitica* *Aphanomyces stellatus* *Dictyuchus sterilis* *Leptomitus lacteus* (*)	Poland	[40]	
*Rana temporaria*	LC, 2008	Stable	Eggs (l)	“*Saprolegnia diclina*-*S. parasitica* complex”	UK	[47]	
*Rana temporaria*	LC, 2008	Stable	Eggs (w)	*Saprolegnia* spp. (s)	Scotland	[70]	
*Scinax garbei*	LC, 2004	Stable	Eggs (n.d)	*Saprolegnia* sp. 2 (s)	Ecuador	[36]	KF718071 KF718072
*Spea bombifrons*	LC, 2015	Stable	Tadpoles (w)	*Saprolegnia* sp.	USA	[71]	
*Xenopus laevis*	LC, 2020	Increasing	Adult (c)	*Saprolegnia* sp.	USA	[72]	
*Xenopus laevis*	LC, 2020	Increasing	Embryo (l)	*S. hypogyna* (s) *S. delica* (s) *S. diclina* (s) *S. parasitica* (N12) (s) *S. ferax* (SAP442) (s)	Scotland	[15]	KF420214 KF420220 KF420212/KF420236 FN186030 AM228845
*Xenopus longipes*	CR, 2017	Decreasing	Adults (w)	“Saprolegniosis”	Cameroon	[24]	
**Urodela**							
*Ambystoma gracile*	LC, 2015	Stable	Larvae (l)	*Saprolegnia* sp.	USA	[55]	
*Ambystoma gracile*	LC, 2015	Stable	Egg (w)	*Saprolegnia ferax* (s) *Saprolegnia* sp. (s)	USA	[31]	EU124763 EU124752
*Ambystoma macrodactylum*	LC, 2015	Stable	Eggs (w)	*Saprolegnia* sp. (s) *Leptolegnia* sp. (s)	USA	[61]	
*Ambystoma maculatum*	LC, 2015	Stable	Eggs (w) (l)	*Saprolegnia* sp. *Achlya* sp.	USA	[41]	
*Ambystoma maculatum*	LC, 2015	Stable	Eggs (w) (l) Embryos (w) (l)	*Saprolegnia* (s)	USA	[66]	EU480454
*Ambystoma maculatum*	LC, 2015	Stable	Larvae (l)	*Saprolegnia* sp.	USA	[73]	
*Ambystoma maculatum*	LC, 2015	Stable	Eggs (w) (l)	*Saprolegnia torulosa* (s) *S. diclina* (s) *S. turfosa* (s) *Achlya papilosa* (s)	USA	[74]	KC758888 to KC758895
*Ambystoma talpoideum*	LC, 2004	Stable	Eggs (w) (l)	“Water mold infection”	USA	[75]	
*Ambystoma tigrinum*	LC, 2015	Stable	Larvae (l)	*Saprolegnia*	USA	[76]	
*Andrias davidianus*	CR, 2004	Decreasing	Adult (c)	Saprolegniosis (**)	USA	[25]	
*Andrias japonicus*	CR, 2004	Decreasing	Eggs (c)	“Water mold infection”	Japan	[77]	
*Hynobius dunni*	VU, 2021	Decreasing	Eggs (c)	Saprolegniosis (**)	UK	[26]	
*Notophthalmus viridescens*	LC, 2015	Stable	Adult (w)	*Achlya* sp. (s)	USA	[57]	GU014277
*Triturus vulgaris* ^14^	LC, 2009	Stable	Eggs (l)	*Saprolegnia ferax* *Saprolegnia parasitica* *Aphanomyces stellatus* *Dictyuchus sterilis* *Leptomitus lacteus* (*)	Poland	[40]	
*Triturus vulgaris* ^14^	LC, 2009	Stable	Eggs (l) Adults (l)	*Saprolegnia* sp.	UK	[78]	

(*) Other species of *Saprolegnia* sp., *Achlya* sp. were identified, but these two appeared in all the amphibian species analyzed. (**) Saprolegniosis agent not confirmed as *Saprolegnia* sp. Note: Since cited publications, some species were reclassified in relation to their original publication. We next list the changes in the present order: name on the publication—actual name used based on AmphibiaWeb site [79]. 1—*Bufo americanus—Anaxyrus americanus*; 2—*Bufo boreas*—*Anaxyrus boreas boreas* or *A. b. halophilus;* 3*—Bufo bufo—Bufo bufo* or *B. spinosus;* 4—*Bufo calamita—Epidalea calamita;* 5*—Bufo marinus—Rhinella marina;* 6*—Hyla regilla—Pseudacris regilla* or *Hyla wrightorum;* 7*—Lithobates berlandieri*—*Rana berlandieri;* 8*—Lithobates catesbeiana*—*Rana* *catesbeiana;* 9—*Rana esculenta—Pelophylax ridibundus* or *P. lessonae;* 10—Rana kl. *Esculenta—Pelophylax kl. esculentus;* 11—*Rana lessonae*—*Pelophylax lessonae;* 12—*Rana plancyi chosenica*—*Pelophylax chosenicus*; 13—*Rana ridibunda*—*Pelophylax ridibundus*; 14—*Triturus vulgaris*—*Lissotriton vulgaris*.

**Table 2:** Summary table of published works on compounds that are used to treat saprolegniosis in amphibians, not considering the pathogen agent specifically. Potential new treatments tested in vitro, though not yet in use, are also reported. Note: The following table is shown for informational purposes only, and any treatment requirements should not be determined without the consultation of a specialized veterinarian.

Treatment Agent	Amphibians		Reference
**Malachite green ^†1^**	0.2 mg/L as a bath for 1 h daily 67 mg/L for 15 s, once daily for 2–3 days		[114] [100]
**Formalin ^†2^**	1.5 mL/L of a 10% formalin solution dip for 10 min (48 h)		[115,116]
**Methylene blue**	50 mg/mL 10-s dip, <2 mg/mL for tadpoles		[114] [115]
**Copper sulphate**	500 mg/L for 2 min, once daily for 5 days, then once weekly until healed)		[100]
**Sea salt**	Bath:10–25 g/L for 5–30 min		[114]
**Sodium chloride**	Bath:10–25 g/L for 5–30 min		[114]
**Calcium propionate**	Solution of 2–3% calcium propionate for 1 min, daily		[117]
**Potassium permanganate**	200 mg/L for 5 min 1:5000 for 5 min		[100] [118]
**Mercurochrome**	2% solution, topical painting		[117]
**Benzalkonium chloride**	2 mg/L as a 60-min bath (24 h) 0.25 mg/L, 3 times a week		[100] [116]
**New therapies**			
**Treatment agent**	**Species tested**	**In vitro**	**Reference**
**Boric acid**	*S. parasitica* *S. diclina*	800 mg/L (complete inhibition) >200 mg/L ↓ spore activity and mycelial growth	[119,120]
**Clotrimazole**	*S. parasitica* *S. diclina* *S. ferax*	MIC_100_ of 1 to 2 mg/L	[121]
**Virkon-S**	*S. parasitica*	Spore and mycelial growth inhibition >4 mg/L	[122]
**Essential oils**	*S. parasitica* *S. diclina* *S. ferax* *S. australis*	Essential oils and pure compounds (See references)	[123,124,125,126,127,128,129]

^†1^ Banned internationally since 2002 due its carcinogenic and toxicological effects. ^†2^ Potential toxicity to handlers and danger to the ecosystem. Formalin also has been banned in some countries.

## Data Availability

The data presented in this study are available on request from the corresponding author.

## Supplementary Materials

The following are available online at https://www.mdpi.com/article/10.3390/jof8050537/s1, Table S1: sequences used in the phylogenetic tree construction and suggested names. Table S2: Raw data about geographic distribution of saprolegniosis reports in wild populations, Table S3: Species reports count. Figure S1: Total phylogenetic ITS tree.
