# Supplementary material for: Saprolegniosis in Amphibians: An Integrated Overview of a Fluffy Killer Disease
Source: J Fungi (Basel). 2022 May 22;8(5):537. doi: 10.3390/jof8050537 (PMC9144230; doi:10.3390/jof8050537)

Table S1 – ITS sequences relatively to *Saprolegniales* found associated to amphibians. Bolt names correspond to the suggested sequence after the phylogenetic analysis of the sequences compared with the suggested MOTUS in Sandoval-Sierra review.

**Accession Number**	**Sequences used on the tree**	**MOTU/Confirmed name**	**Reference**
**EU348372**	*Saprolegnia* sp.	***Saprolegnia aenigmatica***	67
**EU348371**	*Saprolegnia* sp.	***Saprolegnia aenigmatica***	67
**AM228837**	*Saprolegnia australis*	*Saprolegnia australis*	66
**AM228848**	*Saprolegnia diclina*	*Saprolegnia diclina*	66
**EU071706**	*Leptolegnia* sp.	***Saprolegnia aenigmatica***	59
**EU071707**	*Saprolegnia* sp.	***Saprolegnia ferax***	59
**EU480454**	*Saprolegnia* sp.	***Saprolegnia aenigmatica***	65
**AM228844**	*Saprolegnia diclina*	*Saprolegnia diclina*	48
**AM228845**	*Saprolegnia ferax*	*Saprolegnia ferax*	48
**JQ974983**	*Saprolegnia ferax*	*Saprolegnia ferax*	45
**JQ974984**	*Saprolegnia ferax*	*Saprolegnia ferax*	45
**JQ974985**	*Saprolegnia* sp.	***Saprolegnia aenigmatica***	45
**JQ974986**	*Saprolegnia* sp.	***Saprolegnia aenigmatica***	45
**JQ974987**	*Saprolegnia* sp.	***Saprolegnia aenigmatica***	45
**JQ974988**	*Saprolegnia* sp.	***Saprolegnia aenigmatica***	45
**JQ974989**	*Achlya treleaseana*	***Aplanes sp.***	45
**JQ974990**	*Achlya oligacantha*	***Newbya sp.***	45
**JQ974991**	*Achlya* sp.	***Newbya sp.***	45
**JQ974992**	*Achlya* sp.	***Aplanes sp.***	45
**JQ974993**	*Saprolegnia* sp.	***Saprolegnia* sp. 6**	45
**JQ974994**	*Saprolegnia* sp.	***Saprolegnia* sp. 6**	45
**JQ974995**	*Saprolegnia* sp.	***Leptolegnia* sp.**	45
**JQ974996**	*Saprolegnia* sp.	***Leptolegnia* sp.**	45
**JQ974997**	*Saprolegnia* sp.	***Leptolegnia* sp.**	45
**JQ974998**	*Saprolegnia anisospora*	*Saprolegnia anisospora*	45
**JQ974999**	*Saprolegnia torulosa*	*Saprolegnia torulosa*	45
**AM228845**	Saprolegnia *ferax*	*Saprolegnia ferax*	72

**FN186030**	*Saprolegnia parasitica*	*Saprolegnia parasitica*	72
**KF420212**	*Saprolegnia diclina*	*Saprolegnia diclina*	72
**KF420214**	*Saprolegnia hypogyna*	***Saprolegnia parasitica***	72
**KF420220**	*Saprolegnia delica*	*Saprolegnia delica*	72
**KF420236**	*Saprolegnia delica*	*Saprolegnia delica*	72
**KC758888**	*Saprolegnia diclina*	*Saprolegnia diclina*	74
**KC758889**	*Saprolegnia diclina*	*Saprolegnia diclina*	74
**KC758890**	*Saprolegnia torulosa*	*Saprolegnia torulosa*	74
**KC758891**	*Saprolegnia torulosa*	*Saprolegnia torulosa*	74
**KC758892**	*Saprolegnia torulosa*	*Saprolegnia torulosa*	74
**KC758893**	*Saprolegnia torulosa*	*Saprolegnia torulosa*	74
**KC758894**	*Achlya papilosa*	***Aplanes sp.***	74
**KC758895**	*Saprolegnia turfosa*	*Saprolegnia turfosa*	74
**MK372991**	*Saprolegnia ferax*	*Saprolegnia ferax*	49
**MK046073**	*Saprolegnia australis* SC1	*Saprolegnia australis* SC1	In press
**GU014271**	*Achlya* sp.	***Saprolegnia aenigmatica***	56
**GU014279**	*Leptolegnia* sp.	***Saprolegnia* sp.**	56
**GU014277**	*Leptolegnia* sp.	***Saprolegnia* sp.**	56
**GU014283**	*Saprolegnia* sp.	***Saprolegnia ferax***	56
**EU124746**	*Saprolegnia anisospora*	*Saprolegnia anisospora*	31
**EU124747**	*Saprolegnia anisospora*	*Saprolegnia anisospora*	31
**EU124748**	*Saprolegnia anisospora*	*Saprolegnia anisospora*	31
**EU124749**	*Saprolegnia* sp.	***Leptolegnia* sp.**	31
**EU124750**	*Saprolegnia* sp	***Leptolegnia* sp.**	31
**EU124751**	*Saprolegnia* sp.	***Leptolegnia* sp.**	31
**EU124752**	*Saprolegnia* sp.	***Leptolegnia* sp.**	31
**EU124753**	*Saprolegnia* sp.	***Leptolegnia* sp.**	31
**EU124754**	*Saprolegnia* sp.	***Leptolegnia* sp.**	31
**EU124755**	*Saprolegnia* sp.	***Leptolegnia* sp.**	31
**EU124756**	*Saprolegnia* sp.	***Leptolegnia* sp.**	31
**EU124757**	*Saprolegnia* sp.	***Leptolegnia* sp.**	31
**EU124758**	*Saprolegnia* sp.	***Leptolegnia* sp.**	31

**EU124759**	*Saprolegnia* sp.	***Leptolegnia* sp.**	31
**EU124760**	*Saprolegnia* sp.	***Leptolegnia* sp.**	31
**EU124761**	*Saprolegnia* sp.	***Saprolegnia* sp. 6**	31
**EU124762**	*Achlya stellata*	***Newbya sp.***	31
**EU124763**	*Saprolegnia ferax*	*Saprolegnia ferax*	31
**EU124764**	*Saprolegnia diclina*	*Saprolegnia diclina*	31
**EU124765**	*Saprolegnia diclina*	*Saprolegnia diclina*	31
**EU124766**	*Saprolegnia cf. kauffmanniana*	***Saprolegnia aenigmatica***	31

Table S2- Data information used in the wild saprolegniasis report map location (Figure 1). ID Correspond to the identification of the points in the map. ***m.a.s.l.***- meters above sea level.

Country	Reference	ID	Latitude	Longitude	Altitude (m.a.s.l)
USA	Bragg & Bragg, 1958	1	35.2037	-97.1715	
USA	Bragg, 1962	2	35.2037	-97.1715	
Netherlands	Strijbosch, 1979	3	51.8000	5.8000	
Netherlands	Leuven *et al.*, 1986	4	53.3865	5.2365	
Netherlands	Leuven *et al.*, 1986	4	51.3662	5.7056	
Netherlands	Leuven et al. 1986	4	51.4891	4.3328	
UK	Banks & Beebee, 1988	5	54.3673	-3.4495	
UK	Banks & Beebee, 1988	5	51.0863	-0.8660	
UK	Beattie *et al.*, 1991	6	54.7786	-2.0860	183 to 617
USA	Blaustein *et al.*, 1994	7	45.4969	-121.8194	1220
Australia	Williamson & Bull, 1994	8	-35.1230	138.7292	
USA	Kiesecker & Blaustein, 1997	9	45.4969	-121.8194	1220
USA	Kiesecker & Blaustein, 1997	9	44.1025	-121.6259	2000
USA	Kiesecker & Blaustein, 1997	9	44.0321	-121.6858	2000
USA	Kiesecker & Blaustein, 1997	9	44.0903	-121.7362	2600
USA	Kiesecker & Blaustein, 1997	9	44.2146	-121.8904	1500
USA	Kiesecker & Blaustein, 1997	9	44.5319	-122.0778	1190
USA	Kiesecker & Blaustein, 1997	9	43.9252	-121.7800	1190
Australia	Berger *et al.*, 2001	10	-19.2329	146.7809	1150
Australia	Berger *et al.*, 2001	10	-19.6101	146.9617	1150
USA	Kiesecker *et al.*, 2001	11	44.5324	-122.0777	
USA	Gomez-Mestre *et al.*, 2006	12	42.4925	-70.9775	
USA	Johnson *et al.*, 2008	13	47.3105	-121.2997	
USA	Petrisko *et al.*, 2008	14	46.1013	-114.2616	
USA	Petrisko *et al.*, 2008	14	45.1067	-116.0694	
USA	Petrisko *et al.*, 2008	14	47.4682	-121.4501	
USA	Ruthig, 2008	15	33.3293	-81.7235	
Korea	Kim *et al.*, 2008	16	36.1590	127.7302	
Spain	Fernández-Benéitez *et al.*, 2008	17	40.2950	-5.1044	
Sweden	Sagvik *et al.*, 2008a, 2008b	18	57.7500	11.8000	
Sweden	(plus, same points as Uller 2009)	18	57.6692	11.9517	
USA	Ruthig, 2009	19	37.3745	-80.5226	

Sweden	Uller *et al.,* 2009	20	57.7333	11.6667	
Sweden	Uller *et al.*, 2009	20	57.7167	11.6333	
Sweden	Uller *et al.*, 2009	20	55.7000	13.3500	
Sweden	Uller *et al.*, 2009	20	55.5500	13.6333	
USA	Karraker & Ruthig, 2009	21	43.9743	-74.1875	
Camaroon	Blackburn *et al.*, 2010	22	6.2000	10.4500	2227
Colombia	Prada-Salcedo *et al.*, 2010	23	6.0306	-73.1499	2900
Spain	Fernández-Benéitez *et al.*, 2011	24	40.2694	-5.2464	1927
Spain	Fernández-Benéitez *et al.*, 2011	24	40.3631	-5.6097	1614
USA	Ault *et al.*, 2012	25	47.3106	-121.2997	
USA	Ruthig & Provost-Javier, 2012	26	37.3749	-80.5224	
Argentina	Perotti *et al.*, 2013	27	-41.0833	-71.4500	780
Argentina	Perotti *et al.*, 2013	27	-41.1167	-71.3667	
USA	Croshaw, 2014	28	33.8085	-81.5829	
Scotland	Muir *et al.*, 2015	29	55.9825	-4.0059	179
Scotland	Muir *et al.*, 2015	29	55.8331	-4.2739	163
Scotland	Muir *et al.*, 2015	29	55.8041	-4.3519	149
Scotland	Muir *et al.*, 2015	29	55.9844	-4.0060	72
Scotland	Muir *et al.*, 2015	29	56.0263	-4.1134	228
Scotland	Muir *et al.*, 2015	29	56.5002	-4.2354	215
Scotland	Muir *et al.*, 2015	29	56.5423	-4.2291	990
Scotland	Muir *et al.*, 2015	29	56.4994	-4.2523	223
Scotland	Muir *et al.*, 2015	29	56.5188	-4.2958	900
Scotland	Muir *et al.*, 2015	29	56.2046	-4.7628	155
USA	Urban *et al.,* 2015	30	41.4145	-72.7008	
Korea	Groffen *et al.*, 2019	31	37.8678	126.8646	
Korea	Groffen *et al.*, 2019	31	36.4859	127.7378	
Korea	Groffen *et al.*, 2019	31	36.0822	127.4950	
Korea	Groffen *et al.*, 2019	31	37.6664	128.8459	
Korea	Groffen *et al.*, 2019	31	37.0614	128.2560	
Korea	Groffen *et al.*, 2019	31	36.3447	128.2489	
Korea	Groffen *et al.*, 2019	31	34.9914	127.3640	
USA	Sadinski, Gallant, & Cleaver, 2020	32	37.9531	-119.2791	2800 to 3200
Portugal	Costa *et al.* in prep	33	40.6348	-8.6587	

Table S3- Species reported and number of reports of saprolegniasis in the wild. Data counts from literature review.

Species	Reports in wild
Anaxyrus canorus	1
Atelopus mittermeieri	1
Bombina bombina	-
Bufo americanus	1
Bufo boreas	5
Bufo bufo	2
Bufo calamita	5
Bufo gargarizans	1
Bufo marinus	1
Crinia signifera	1
Elachistocleis bicolor	-
Engystomops petersi	-
Hyla molleri	-
Hyloscirtus alytolylax	-
Lithobates berlandieri	1
Lithobates catesbeianus (Rana catesbeiana)	2
Pelobates cultripes	1
Pelobates fuscus	1
Pelophylax perezi	1
Physalaemus albonotatus	-
Pleurodema thaul	1
Pseudacris crucifer	2
Pseudacris regilla (Hyla regilla)	4
Pseudacris streckeri	1
Rana arvalis	5
Rana aurora	2
Rana cascadae	4
R. clamitans	1
R. dalmatina	-
R. esculenta	2
Rana huanrenensis	1
Rana lessonae	-
Rana luteiventris	1
Rana plancyi chosenica	-
Rana pretiosa	1
Rana ridibunda	-
Rana sphenocephala	1

Rana sylvatica	2
Rana temporaria	4
Scinax garbei	-
Spea bombifrons	1
Xenopus laevis	-
Xenopus longipes	1
Ambystoma gracile	1
Ambystoma macrodactylum	1
Ambystoma maculatum	3
Ambystoma talpoideum	1
Ambystoma tigrinum	-
Andrias davidianus	-
Andrias japonicus	-
Hynobius dunni	-
Notopthalmus viridescens	1
Triturus vulgaris	-

Figure S1. Maximum likelihood phylogram depicting phylogenetic relationships inferred among 167 isolates, based on combined analysis of ITS DNA sequence data. The terminal number correspond to the bibliographic reference whose sequence belongs in Table 1, SAPxxx coded samples belong to Sandoval-Sierra review (associated to amphibians) as well MOTUs. The other sequences with reference name belong to Magray et al. review. Notice that the circle identifies sequences that were included on ITS tree of Magray's article, and in the present tree are highlighted to show that it is wrong, shouldn't be included in this analyses because they are not ITS. Bootstrap values (>50%) are presented to the right of each node.

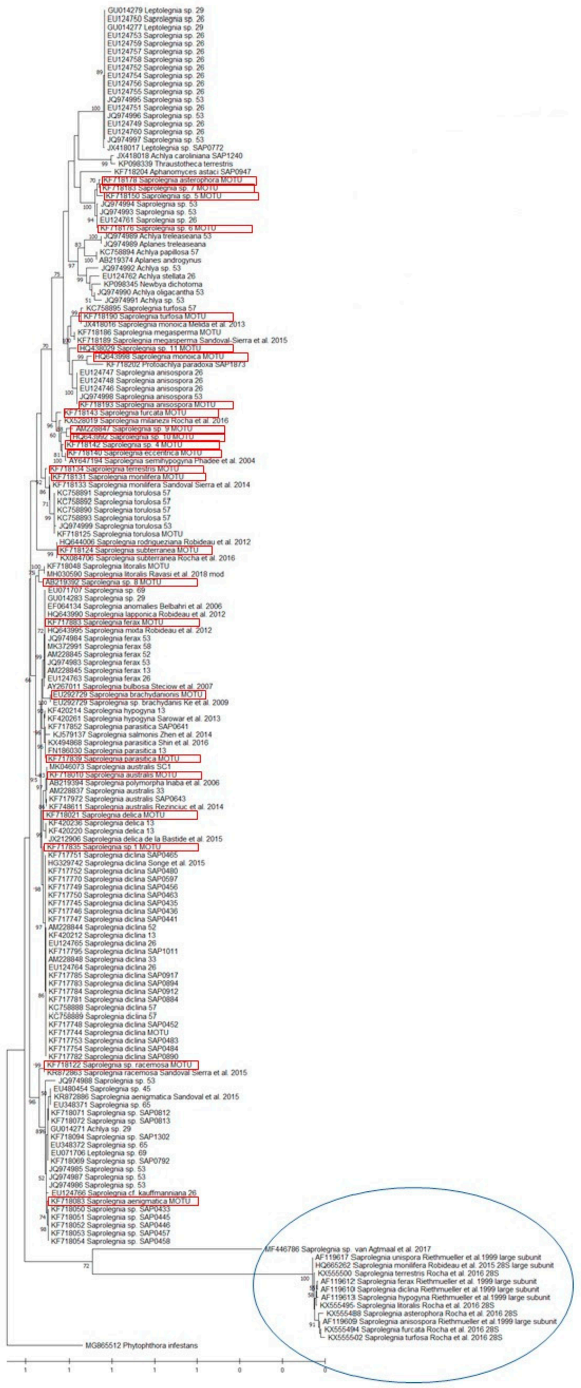

Supplement: Supplementary file 1 [file jof-08-00537-s001.zip › jof-1574694-SI.pdf]
